# Current Mechanistic and Pharmacodynamic Understanding of Melanocortin-4 Receptor Activation

**DOI:** 10.3390/molecules24101892

**Published:** 2019-05-16

**Authors:** Shubh Sharma, Alastair S. Garfield, Bhavik Shah, Patrick Kleyn, Ilia Ichetovkin, Ida Hatoum Moeller, William R. Mowrey, Lex H.T. Van der Ploeg

**Affiliations:** Rhythm Pharmaceuticals, Boston, MA 02116, USA; ssharma@rhythmtx.com (S.S.); agarfield@rhythmtx.com (A.S.G.); bshah@rhythmtx.com (B.S.); pkleyn@rhythmtx.com (P.K.); ichetov@rhythmtx.com (I.I.); imoeller@rhythmtx.com (I.H.M.); bmowrey@rhythmtx.com (W.R.M.)

**Keywords:** melanocortin-4 receptor, obesity, peptide agonist, cardiovascular profile, GαS signaling, receptor desensitization, receptor internalization

## Abstract

In this work we summarize our understanding of melanocortin 4 receptor (MC4R) pathway activation, aiming to define a safe and effective therapeutic targeting strategy for the MC4R. Delineation of cellular MC4R pathways has provided evidence for distinct MC4R signaling events characterized by unique receptor activation kinetics. While these studies remain narrow in scope, and have largely been explored with peptidic agonists, the results provide a possible correlation between distinct ligand groups and differential MC4R activation kinetics. In addition, when a set of small-molecule and peptide MC4R agonists are compared, evidence of biased signaling has been reported. The results of such mechanistic studies are discussed.

## 1. Introduction

It is with great pleasure we dedicate this review article to Prof. Victor J. Hruby, in honor of his 80th birthday. Prof. Hruby has been a seminal leader in several areas of peptide research. His research has blazed the trail for melanocortin research efforts, including building understanding of chemistry, biology and pharmacology of melanocortins. Prof. Hruby has made many pivotal contributions to the scientific community, by creating widely used melanocortin receptor agonists, MT-I (NDP-α-MSH), MT-II, and the melanocortin receptor-3 and -4 antagonist SHU9119. These reagents have been instrumental in unraveling the function of melanocortin receptors including their roles in pigmentation, energy homeostasis, body weight regulation and sexual arousal. Prof. Hruby continues to make powerful contributions to the melanocortin research field, through the development of potent and selective agonists and antagonists for various melanocortin receptor subtypes.

The central hypothalamic melanocortin-4 receptor (MC4R) is a uniquely validated therapeutic target for the treatment of obesity based on both pharmacologic and human genetic evidence [1,2,3,4]. Acting in concert with leptin (a satiety hormone), ghrelin (a hunger hormone) and their receptors, the MC4R holds a key position in the regulation of energy homeostasis and body weight. The MC4R and leptin receptor are key components of the MC4R pathway, which, when disrupted by genetic defects in any of these contributing receptor/ligand systems, causes impaired energy balance [1,5,6,7]. A variety of peptide and small molecule MC4R agonists have been developed over the past nearly three decades and have been shown in rodent models to elicit decreases in food intake and body weight. However, the diverse nature of MC4R-driven pharmacological efficacy has posed challenges in developing an MC4R agonist for the treatment of obesity. These hurdles include MC4R-related sympathetic activation leading to elevation of blood pressure (BP) and heart rate (HR), as well as activation of sexual arousal [8,9,10,11,12]. As a result, the feasibility of targeting the MC4R for treating obesity by peptides and small molecules ligands has been called into question, despite intense drug discovery and development activity which started in the 1990s. There have been some notable successes in creating MC4R agonist compositions, including orally bioavailable leads (for example, Merck compounds MK-0493 and MB243; Pfizer compound-13; and several Neurocrine NBI compounds described in MacNeil et al. [13]; Palucki et al. [14]; Ujjainwalla and Sebhat [15]; Chen et al. [16]; Krishna et al. [17]; He et al. [18]; and Lansdell et al. [19] and reviewed in Todorovic and Haskell-Luevano [20] and Ericson et al. [21]. MK-0493 evaluated in a phase-1 human study was shown to be ineffective in controlling food intake or body weight meaningfully [17]. A few peptide MCR agonist compositions, including LY2112688, MC4R-NN2-0453, and AZD2820, were also explored in early clinical studies for the treatment of obesity (Table 1). However, their development was stopped due to several adverse effects, including increased HR and BP, hyperpigmentation (melanocortin receptor-1 (MC1R)-driven), and sexual arousal, which were seen in early clinical trials. Similarly, development of bremelanotide, an MC4R peptide agonist for the treatment of male erectile dysfunction, was halted following adverse effects, including BP and HR elevation, as well as nausea and vomiting [22]. However, bremelanotide is currently being investigated for the treatment of hypoactive sexual dysfunction in pre-menopausal women [23].

Setmelanotide, an eight amino acid cyclic MC4R agonist peptide (Table 1), is being investigated in several clinical studies for the treatment of obesity, including rare genetic disorders of obesity. These genetic deficiencies include subjects with pro-opiomelanocortin (*POMC*) deficiency, proprotein-convertase (*PCSK1*) deficiency, leptin receptor (*LEPR*) deficiency, Prader-Willi syndrome (*PWS*), Bardet-Biedl syndrome (*BBS*), Alström syndrome (*AS*), and selected other genetic forms of early-onset severe obesity arising from defects that impair the MC4R pathway. Earlier results indicate that setmelanotide is generally well tolerated in humans and holds promise for treating obesity and hyperphagia in subjects with these rare genetic disorders of obesity.

This review compares the unique pharmacological and mechanistic profiles of several MC4R agonists based on a series of in vivo and in vitro studies. Special emphasis is placed on comparative pharmacological data obtained for the MC4R agonist peptides setmelanotide and LY2112688 [11]. While both setmelanotide and LY2112688 are potent MC4R agonists which can elicit decreases in food intake and body weight in animal models, only setmelanotide lacks adverse increases in cardiovascular activity (HR and BP) in non-human primates and humans [3,24,25]. Studies highlighting key differentiating features among these two agonists are discussed.

## 2. Agonist Induced Signaling, Desensitization, and Internalization of MC4R

Recent investigations which may help differentiate various in vivo pharmacological profiles of certain MC4R agonists have revealed an agonist-based bias for the activation of distinct MC4R intracellular signaling pathways. Studies probing differential MC4R signaling have included assays performed under equilibrium ligand binding conditions or temporally dynamic activation of the MC4R followed by the measurement of their impact on internalization and desensitization of the MC4R. Various intracellular pathways that could be invoked in neural MCR signaling, along with biased signaling ligands, have recently been reviewed by Yang and Tao [26]. Various signaling pathways explored with MC4R include the use of different G-proteins, including Gαs, Gαi/Gαo, Gαq, and G-protein dependent mitogen-activated protein kinases/extracellular signal-regulated kinases (MAPK/ERK) activation, as well as G-protein independent effects on the potassium channel Kir7.1 on MC4R neurons. These are discussed in more detail in the following sections.

The most ubiquitous MC4R signaling events described are mediated through Gαs activation, leading to the production of intracellular cyclic adenosine monophosphate (cAMP). However, recent studies using surrogate reporter systems in MC4R-expressing cells have produced indirect evidence for the involvement of a Gαq signaling pathway leading to the activation of phospholipase-C (PLC) and calcium mobilization [4]. Some investigators have explored the involvement of the Gi/o-mediated pathway, without, however, clear evidence of its involvement [27]. This initial evidence of possible divergence in signaling pathways downstream of MC4R is of interest and requires in depth follow-up to understand its impact in physiologically relevant signaling events. The results of these studies are summarized here and include evaluations of setmelanotide, a related compound RM-511 [28], and the LY2112688 MC4R agonist peptide.

### 2.1. Equilibrium Binding and Activation

It is well established that MC4R signals through Gαs under equilibrium ligand binding conditions using human embryonic kidney (HEK), Chinese hamster ovary (CHO), or monkey kidney tissue derived fibroblast-like cell (COS) cell-based systems stably transfected with MC4R [3,4,14,19,24,28,29,30,31]. Under these equilibrium conditions, receptor activation by both peptide and small molecule agonists can be antagonized by MC4R antagonists like SHU9119 or Agouti gene-related peptide (AgRP). While AgRP is an endogenous antagonist of MC4R mediated GαS signaling, it has also been shown in in vitro systems to function as an inverse agonist by decreasing basal levels of cAMP [32,33]. Table 2 summarizes the effective concentration for 50% stimulation (EC_50_) values for cAMP stimulation and Ki (binding constant) values for receptor binding for setmelanotide, LY2112688, Melanotan-II (MT-II) and alpha-melanocyte stimulating hormone (α-MSH, an endogenous agonist) in CHO cells stably transfected with human (h)MC1R, hMC3R, hMC4R, or hMC5R (these agonists do not interact with the MC2R). In these experiments, setmelanotide, MT-II, and LY2112688 displayed approximately similar binding affinities for the MC4R. In cAMP functional assays performed under equilibrium conditions, the potencies for each of these three agonists were also equivalent when compared between setmelanotide and LY2112688 or MT-II, with setmelanotide being about three times less active.

### 2.2. Dynamic Signaling upon Exposure to MC4R Agonists

In prior studies using time-resolved signaling conditions, LY-2112688, MT-II, THIQ, and a setmelanotide analog, RM-511, were shown to differentiate in their dynamic effects during MC4R signaling or antagonism by AgRP [34].

These studies by Molden et al. [34] employed acute exposure to the MC4R agonist and applied cell-based reporter moiety and receptor tags for tracking receptor activation kinetics. Using a temporally-resolved Förster resonance energy transfer (FRET) based cAMP assay with Neuro2A_HA-MC4R-GFP_ cells (stably expressing epitope-tagged hemagglutinin-MC4R-green fluorescent protein along with endogenous MC4R), Molden et al. [34] revealed evidence of biased signaling dynamics with this set of MC4R agonists, as measured by Gαs mediated cAMP induction. These studies were performed at ≥10 fold the respective EC_50_ concentrations of the agonists and investigated agonist stimulated receptor internalization and desensitization. In these assays, cAMP induction by either α-MSH (200 nM) or LY2112688 (100 nM) could be quickly reversed upon withdrawal of the ligand from the incubation medium and washing the cells free of ligand. These observations contrasted with the remarkable robust stimulation of signaling by MT-II (2 nM), THIQ (1 μM), or RM-511 (100 nM), which persisted for the 1 hr observation period following withdrawal of the respective agonists from the medium and washing of the cells. Furthermore, continued MT-II-induced stimulation could not be reversed by treatment with AgRP. The prolonged non-antagonizable cAMP stimulation may have been due to the presence of internalized constitutively active MC4R. Agonist-induced internalization of MC4R was further studied with an α-MSH (500 nM) re-challenge following initial ligand exposure and wash off (Table 3). In this experiment the cells were treated with the MC4R agonist for 10 min, followed by its withdrawal (washing off) and immediate re-challenge with α-MSH (500 nM) to observe the kinetics of re-accumulation of cAMP. After the wash off, the cAMP stimulation initiated by both α-MSH and LY2112688 was fully reversed, while THIQ stimulation was only partially reversed. The stimulation initiated by MT-II and RM-511 could not be reversed upon washing. Therefore, both MT-II and RM-511 were argued to induce receptor internalization and continued signaling, while α-MSH and LY2112688 were ineffective, and THIQ was able to induce this only partially. Interestingly, Garnell et al. [35] have previously shown that intracellularly targeted α-MSH to intracellular MC4R at the endoplasmic reticulum (by co-transfecting both in neuroblastoma N2A cells) can lead to cAMP stimulation. These activated MC4Rs do not desensitize and can cycle to the cell surface. The level of this intracellular MC4R stimulation is comparable to cAMP activation achieved by acute exposure of the MC4R transfected N2A cells to α-MSH.

Earlier investigations of agonist-induced time-dependent internalization of MC4R expressed in HEK cells and COS cells have also been reported by Shinyama et al. [29]. However, unlike the acute exposure studies of Molden et al. [34], the studies of Shinyama et al. [29] reported that chronic exposure to α-MSH (0.1–1000 µM) in GT1-7 mouse hypothalamic cells caused loss of MC4R activity. This desensitization was interpreted to be due to MC4R internalization in the absence of constitutive signaling. This conclusion was based on the observation that impaired cAMP stimulation resulted after the washing off of the agonist, followed by a re-challenge with 100 µM α-MSH. Useful mechanistic insights on receptor internalization have also been developed. Shinyama et al. [29] found that internalization of the MC4R was dependent on β-arrestin and dynamin, as well as partly dependent on protein kinase-A (PKA) activation. Using a specific phosphorylation inhibitor, H89, the authors showed that Thr312 and Ser329/330 residues in the c-terminal tail of the MC4R are potential phosphorylation sites upon activation of the receptor by α-MSH. These phosphorylation sites were observed to be critical for the recruitment of β-arrestin and subsequent internalization of the receptor. Based on these studies, Shinyama et al. [29] concluded that agonist-induced phosphorylation at the c-terminus of the MC4R is manifested by an agonist-induced conformational change in the receptor needed for its internalization. For example, forskolin increased cAMP but did not induce receptor internalization [29]. Interestingly, low concentrations of α-MSH causing only a small increase in cAMP were enough to cause acute receptor internalization and desensitization [29]. Based on these results, as well as the observation that haploinsufficiency of MC4R can cause obesity, Shinyama et al. [29] have suggested that the MC4R can be activated following low receptor occupancy, where subtle changes in activation or MC4R numbers may be sufficient to exert control over energy homeostasis. Furthermore, in their studies, AgRP (1–10 nM), an endogenous antagonist of MC4R, caused a concentration-dependent increase in cell surface expression of the MC4R. Synthetic small molecule antagonists are also known to facilitate trafficking and rescue intracellularly held mutated MC4R [36]. Based on these results it was proposed that AgRP may be involved in the cell surface trafficking of the MC4R, whereas the endogenous agonist α-MSH brings about the agonist-induced conformational transition needed for phosphorylation and subsequent internalization and desensitization. Studies by Gao et al. [37] and Cai et al. [38] using MC4R transfected HEK293 cells have provided similar evidence for peptide agonist induced internalization of MC4R with no evidence of associated involvement of PKA, probably due to a different host cell system. Further, peptide antagonists used in these studies failed to induce internalization.

Similar findings have also been reported by Nickolls et al. [31], who studied a set of peptide and non-peptide agonists for cAMP accumulation, calcium mobilization (using a fluorescence imaging plate reader (FLIPR) system), and MC4R internalization (FLAG-tagged MC4R by fluorescence-activated cell sorting) assays. While all the agonists were able to stimulate cAMP accumulation, only the peptidic agonists caused strong responses in the calcium mobilization and internalization assays. In these studies, small molecule agonists were observed to be impaired in both the calcium mobilization and internalization assays (Table 4). This study therefore suggested that the subset of peptide agonists capable of causing internalization were distinct in their signaling mechanism over the non-peptides, which were not able to similarly effect receptor internalization. The peptide agonists included in this study were α-MSH, β-MSH, γ-MSH, des-acetyl-α-MSH, and NDP-α-MSH. The small molecule agonists were THIQ and similarly related analogs, as shown in Table 4.

### 2.3. Evidence for Gαq Signaling

With the widely used cell systems employed for measuring MC4R activation, it has been challenging to show the engagement of other G-protein signaling pathways, including Gαq and Gαi/o. Clément et al. [4] have recently provided evidence using HEK293 cell-based systems that incorporated reporter gene systems, such as cAMP binding response element (CREB) and nuclear factor of activated T-cells (NFAT) reporters, that MC4R agonists can exhibit a differential preference for MC4R signaling through one or the other G-protein coupled pathways. For example, when comparing EC_50_ values of three peptide agonists, α-MSH, setmelanotide, and LY2112688, in the reporter gene-based cAMP (Gαs) and phospholipase C-β(PLC) measured through NFAT reporter assay (a possible surrogate of Gαq and/or Gβγ of Gi/o signaling), interesting observations were made. All the EC_50_ data in this study were obtained under equilibrium stimulation conditions and for the Gαs signaling the EC_50_ values were: α-MSH 23 ± 7 nM; setmelanotide 3.9 ± 1.7 nM, and LY2112688 14 ± 4 nM. In the NFAT signaling assay the EC_50_ values were: α-MSH 480 ± 260 nM; setmelanotide 5.9 ± 1.8 nM; and LY2112688 330 ± 190 nM. While the Gαs signaling (cAMP measurement) among these three ligands was found to be comparable to that observed in the CHO cell-based system with endogenous Gαs-coupled signaling protein, the NFAT reporter system (PLC activity measurement) revealed differences in the ability of these ligands to activate the MC4R measured through NFAT signaling. In this assay, setmelanotide was about 80 times more potent than α-MSH and about 55 times more potent than LY2112688. Clément et al. [4] further provided evidence that the potent setmelanotide induced PLC-β activation through MC4R could be due to Gαq activation, as this effect could not be blocked by pertussis toxin (PTX), which inhibits Gi/o signaling. Li et al. [39] have also shown the presence of MC4R-coupled Gαq/11 signaling in the paraventricular nucleus (PVN) of mice, which upon PLC activation leads to MT-II-induced decrease in feeding without impacting cardiovascular response. It remains to be seen, however, if similar results can be confirmed with a more diverse set of MC4R agonists in similar cell-based assay systems.

Antagonism of setmelanotide-induced cAMP and PLC by AgRP, as seen in a study by Clément et al. [4], provides further evidence for potential differences in the MC4R signaling ability when compared between α-MSH, setmelanotide, and LY2112688. In the cAMP antagonism assay, AgRP was about three times less efficient in inhibiting setmelanotide-related stimulation (AgRP IC_50_ 9.24 nM) than LY2112688 (AgRP IC_50_ 3.56 nM). However, in the PLC assay (through the NFAT reporter gene), 100 nM AgRP could not antagonize setmelanotide-stimulated activation while the stimulation by either α-MSH or LY2112688 was completely antagonized. Therefore, when compared to α-MSH and LY2112688, it was shown that not only can setmelanotide activate the PLC-related signaling pathway more robustly but also that this effect cannot be efficiently antagonized by AgRP.

In summary, the complexity of MC4R intracellular signaling is profound and while the physiological relevance of in vitro identified systems remains to be determined, it seems plausible that agonist-induced MC4R internalization could be critical for MC4R-mediated physiological control in energy homeostasis. It is important to note that the MC4R is proposed to be a constitutively active receptor and its activation at low receptor occupancy with α-MSH is proposed to cause its acute internalization. By contrast, AgRP binds the MC4R and causes its upregulation and translocation to, or retention at, the cell surface. Based on our comparator data, it is possible that the peptidic MC4R agonists capable of inducing MC4R internalization may be considered suitable drug candidates for therapeutic intervention for the treatment of obesity. This finding is of interest as all the small molecules studied were ineffective in causing MC4R internalization. Similarly, selected other drug candidates, including the peptidic lead LY2112688 in comparison to RM-511 and MT-II, activate and desensitize the MC4R differently. While there is no published report on its ability to induce MC4R internalization, one small molecule drug candidate lead, MK0493, was found to be ineffective in inducing weight loss in human clinical evaluation [17].

In addition to the above described G-protein related intracellular signaling of MC4R, there have been reports of additional direct mechanisms for the regulation of MC4R neurons in the PVN of the hypothalamus by G-protein independent mechanisms. For example, Ghamari-Langroudi et al. [40] have indicated the involvement of an inwardly rectifying potassium channel (Kir7.1) that closes upon binding of α-MSH to MC4R to depolarize the MC4R neurons, while the binding of the antagonist AgRP causes hyperpolarization by opening the channel. This α-MSH-induced Kir7.1 signaling was proposed as being central to melanocortin-mediated regulation of energy homeostasis within the PVN, with AgRP acting as an agonist in opening this channel [40]. Additionally, Vongs et al. [41] have shown that NDP-α-MSH can intracellularly activate MAPK/ERK1/2 in MC4R-transfected CHO-K1 cells, an effect that is likely dependent on inositol triphosphate IP3. However, as discussed by Yang and Tao [26], the activation of the MAPK/ERK pathway appears to be much more diverse as, in addition to IP3, it can also be shown to involve PKA and protein kinase-C (PKC)/calcium and to be dependent on the cell systems and the MC4R activation ligand used. Rather than functioning as an antagonist in the GαS-mediated cAMP stimulation, AgRP induced phosphorylation in the ERK pathway. Further studies are needed to sort through the signaling diversity associated with the MAPK/ERK activation pathway.

## 3. Feeding and Weight Loss Studies in Rodents

The efficacy of setmelanotide and other MC4R agonists has been studied in diverse preclinical models, including mouse models of diet-induced obesity (DIO) and Sprague-Dawley rats [28]. In an acute study in DIO mice, 6.4 µmole of setmelanotide (identified in this study as BIM-22493) administered as a single intraperitoneal injection 30 min prior to food presentation strongly and significantly inhibited food intake during refeeding following an overnight fast. These effects appear MC4R-mediated, as in the Kumar et al. study [28] there was no impact on food intake observed in the MC4R-knockout mice, while the MC3R knockout mice responded like wild-type mice, thereby establishing that the setmelanotide effect is dependent on a functional MC4R and does not require MC3R in this model system. Similarly, setmelanotide also reduced food intake during refeeding after an overnight fast in male Sprague–Dawley rats at 100 or 500 nmol/kg administered subcutaneously [28]. Furthermore, in chronic studies performed in DIO mice, setmelanotide (300 nmol/kg/day for 14 days, administered by subcutaneous (SC) osmotic pump) was also shown to reduce body weight and improve glucose homeostasis and dyslipidemia. The suppression of food intake and weight loss was most pronounced during days 1–4 of treatment, and by day 12 the effect on food intake was no longer significant, although food intake still appeared lower compared to controls [28].

In a comparative study with MC4R-homozyogus knockout (MC4R^−/−^), MC4R-heterozygous (MC4R^+/−^), and wild-type mice maintained on a high fat diet (45% kcal fat) and implanted with subcutaneous osmotic pumps containing either 1.34 mg/kg/day setmelanotide or the vehicle, Collet et al. [3] reported that wild-type mice were most sensitive to the treatment emergent weight loss. The MC4R^−/−^ null mice did not respond in this study and the heterozygous MC4R^+/−^ mice exhibited an intermediate response to setmelanotide. This study therefore reinforced the finding that the weight loss effects induced by setmelanotide are fully dependent on the presence of a functional MC4R.

LY2112688 was also shown to be efficacious in a diet-induced obese rat model of food intake and weight loss [30]. In this study, a dose-dependent decrease in cumulative food intake and cumulative body weight loss was observed at doses of 0.075 µmol/kg/day and 0.299 µmol/kg/day administered subcutaneously for 14 days. The impact of the top dose on daily food intake, however, lasted only for the first five days of dosing, as food intake was not different from the vehicle-treated group thereafter. There was also a significant lowering of fat mass seen at the higher dosages, with no impact on lean body mass during this 14-day testing period. Therefore, these MC4R agonists reduce food intake and body weight in rodent models by activating the MC4R, in keeping with the physiological function of the MC4R pathway.

## 4. Body Weight Effects in Obese Non-Human Primates

The effects of SC infusion of setmelanotide (0.50 mg/kg/day for eight weeks) on body weight have been reported for a group of twelve male Rhesus monkeys maintained on high-fat diet (HFD) for approximately 1.5 years before initiation of these setmelanotide dosing studies [24]. The LY2112688 peptide was used in this study as a comparator. Nine of the 12 animals were obese, insulin-resistant, and hypertensive at baseline, and the remaining three animals, classified as diet- resistant, had normal body weight, adiposity, and blood pressure.

After treatment with setmelanotide for one week, there was a significant decrease in food intake (~35%). Similar to the findings in rodents as discussed above, this effect was reported as transient, with food intake normalizing by weeks 4 through 7 of treatment, with a moderate increase in food intake during week 8 of drug treatment. Upon cessation of setmelanotide treatment, a significant increase in food intake was reported over the pretreatment baseline level, followed by normalization of food intake by 12 weeks post-treatment, when the animals had returned to their pretreatment body weight. Interestingly, following cessation of the setmelanotide treatment, body weight remained suppressed for about 2–4 weeks in all study animals before rebounding, as if a post-treatment therapeutic benefit was retained. A significant decrease in food intake was also reported with LY2112688 at the same dose of 0.5 mg/kg/day; however, the decrease was significantly smaller than that seen with a comparable dose of setmelanotide.

Consistent with the significant decrease in food intake, the obese animals were reported to have lost ~0.75 kg (~5% of their body weight) after treatment with setmelanotide for two weeks and ~1 kg after treatment for four weeks. It is interesting to note that even after food intake increased, the animals were reported to continue to lose weight during setmelanotide treatment, leading to a mean peak weight loss of ~13.5%. After cessation of setmelanotide treatment, the animals began to regain body weight (0.5 kg by four weeks post-treatment) and a return to baseline weight by 10–12 weeks post-treatment, by which time food intake had also returned to baseline.

Activation of MC4R pathway has also been associated with several non-feeding related activities, including increased yawning, muscular stiffness, stretching, and penile erections [9]; however, none of these effects were observed in these experiments with setmelanotide.

## 5. Effect of Setmelanotide and LY2112688 on Cardiovascular Function

Comparative cardiovascular effects of setmelanotide and LY2112688 have been reported [24]. Briefly, in this study the groups of obese and lean Rhesus monkeys maintained on an HFD were the same as those used in the weight loss studies described above [24]. The animals received setmelanotide 0.50 mg/kg/day via SC bolus injection for four days, with telemetric measurements of the BP and HR. Following a washout period, the same group of animals received setmelanotide (0.50 mg/kg/day) or LY2112688 (0.17 or 0.50 mg/kg/day) via SC infusion for seven days. Setmelanotide (0.50 mg/kg) administered via an acute SC injection resulted in a mild increase in HR, though the obese Rhesus, in contrast to the rat, exhibited a compensatory drop in BP that persisted for 6–7 h post-dose. Upon subsequent daily bolus SC injections in the Rhesus, the heart rate response was reported to decline on days 2 and 3, and was nearly absent by day 4, while associated reduction in food intake persisted for four days of setmelanotide administrations.

In contrast to the findings reported with setmelanotide administered via SC injection, no acute increase in HR was reported after continuous SC infusion of setmelanotide at 0.50 mg/kg/day for seven days. In fact, a significant decrease in mean HR was reported, primarily in obese animals. HR remained significantly lower in the obese animals four weeks post-dose. Paralleling the decrease in HR, a significant decrease in diastolic BP was also reported in both obese and lean animals. In a follow-up study in the cynomolgus monkey, setmelanotide continuously infused at a 50-fold higher dose of 25 mg/kg/day for three days did not cause any biologically significant changes in systolic pressures, HR, or electrocardiogram (ECG) interval duration or waveforms [25]. By contrast, after SC infusion of LY2112688, increases in BP and HR were observed in the obese Rhesus. Significant mean increases of 15.5 and 16.5 beats per minute (bpm) at 0.17 mg/kg/day of LY2112688 for day 1 and 2, respectively, and 14.4 bpm at 0.5 mg/kg/day on day 2, were observed [24].

In the Sprague-Dawley rat model the HR and mean blood pressure effects of setmelanotide and LY2112688 and diverse other MC4R agonists were studied after single and multiple dose subcutaneous administration, which was were found to induce sustained increases in HR and mean blood pressure [25]. It is therefore interesting to note that while both setmelanotide and LY2112688 induced increased HR and BP in the rat model of cardiovascular (CV) activity, only setmelanotide was devoid of these same effects in the monkey cardiovascular model. The observed effects in the rat model for these MC4R agonists do not differ from previously reported cardiovascular effects revealing increases in blood pressure and heart rate in rodents with the MC4R pan-agonist MT-II [42,43,44]. Interestingly, α-MSH administered intravenously (IV) has been shown to fail to increase HR and BP in mice and rabbits but has been observed to elicit CV effects when administered directly into the circumventricular system, suggesting that limiting central nervous system (CNS) exposure may overcome adverse sympathetic CV sequela in preclinical models [45,46].

## 6. Perspective

MC4R agonists are linked to an increase in sympathetic tone, which is anticipated to directly impact heart rate and mean blood pressure. These effects can be blunted by a co-administration of α and β blockers, outlining the classical activation of sympathetically mediated CV control [10]. Most of these MC4R agonists have been shown to induce increases in mean arterial blood pressure and heart rate, even at anorexigenic doses [47], indicating that MC4R is involved in regulating CV function [9]. The CV activity data obtained to date with setmelanotide and other MC4R agonists suggest a species specific response when compared between rodents and non-human primates. Thus, although an effect on heart rate and blood pressure has been seen in rats, results of studies of non-human primates and CV data obtained for humans [3,24,25] has shown that subcutaneously infused setmelanotide or subcutaneously injected setmelanotide [3] has no effect on heart rate and blood pressure. Furthermore, LY2112688 studied in the same group of Rhesus induced both robust heart rate and blood pressure responses at similar infused dosage levels. These results point to the unique cardiovascular safety of setmelanotide when compared to the LY2112688 peptide. Furthermore, at the same dosage levels, setmelanotide has been associated with a more robust response in weight loss when compared to LY2112688, which is indicative of a favorable therapeutic index for setmelanotide in the treatment of obesity. While the mechanistic reasons for these differences are being studied, earlier studies in rodents have shown that some degree of brain penetration may be needed for the CV response, as shown in studies reporting increased CV effects upon intracerebroventricular (ICV) administration as compared to the IV administration of α-MSH in rodents and rabbits, which has been shown to fail to elicit CV responses [44,45,46]. It is interesting to note that brain penetration of MT-II and other ligands may not be required to elicit beneficial effects in the control of food intake and obesity in rodents [48]. However, exposure below analytically detectable levels of CNS exposure cannot be excluded [48]. Combining these data suggests that apparently poorly brain penetrant MC4R agonists (below analytical detection levels) with a unique receptor activation pharmacology profile may show promise for the treatment of obesity without the CV side effects.

The in vitro MC4R signaling studies discussed show that setmelanotide and LY2112688 diverge in their receptor activation kinetics in transfected MC4R cell reporter systems. In accordance with cell-based observations, we speculate that setmelanotide binding to MC4R is conducive to causing conformational change in the MC4R, which Shinyama et al. [29] have shown to be critical for agonist-induced receptor internalization. Based on the studies summarized above, we speculate that LY2112688, like α-MSH, is not able to trigger this same conformational change and consequently fails to elicit MC4R internalization and constitutive signaling. While the significance of the agonist-induced internalization of the MC4R to its pharmacological profile is yet to be established, additional receptor dynamics, such as homo- or hetero-receptor dimerization states, could be considered as playing a role. In addition, differences in brain penetration or distribution within the brain of each agonist may contribute to the diverse physiological effects observed. For example, it has been reported that the disruption of Gαs signaling, while maintaining Gαq/11 signaling in the PVN of mice, can abolish the cardiovascular effects of pan-agonist MT-II [39]. Furthermore, in humans with gain-of-function MC4R mutations, Lotta et al. [49] have shown that such mutations can present biased MC4R-signaling through MAPK activation and recruitment of β-arrestin, which correlates with their lower body mass index and protection against obesity, type-2 diabetes, and coronary artery disease. Further studies on biased signaling and unique receptor desensitization kinetics of the MCRs are anticipated to provide further insights into the diverse pharmacology of the melanocortin agonists currently in development.

## Figures and Tables

**Table 1:** Structures of various melanocortin-4 receptor (MC4R) agonists evaluated in human clinical studies.

**Setmelanotide:**	Ac-Arg-cyclo(Cys-D-Ala-His-D-Phe-Arg-Trp-Cys)-amide
**LY2112688:**	Ac-D-Arg-cyclo(Cys-Glu-His-D-Phe-Arg-Trp-Cys)-amide
**MC4-NN-0453:**	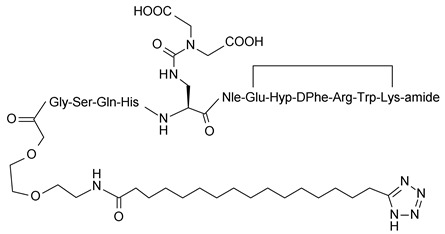
**MK-0493:**	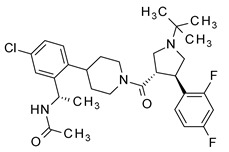
**AZD2820:**	Structure undisclosed

**Table 2:** Inhibitory constants and 50% effective concentrations of peptide agonists in CHO-K1 cells expressing human melanocortin receptors (data from Kievit et al. [24]).

Compound	Binding Assay Ki [nM]				cAMP Assay EC_50_ [nM]			
	hMC1R	hMC3R	hMC4R	hMC5R	hMC1R	hMC3R	hMC4R	hMC5R
α-MSH	0.32	15.5	41.4	332	1.01	1.04	4.7	10.5
MT-II	0.27	24	2.66	23.1	0.2	0.51	0.05	5.33
Setmelanotide	3.9	10	2.1	430	5.8	5.3	0.27	1600
LY2112688	4	35.1	1.84	5160	8.12	10.3	0.09	5760

**Table 3:** Dynamic cAMP stimulation upon ligand incubation, washout, and α-MSH re-challenge, showing persistent signaling by certain MC4R ligands (adapted from Molden et al. [34]).

MC4R Ligands and Incubation Concentration	Dynamic cAMP Response				Comment
	Time = 0 min	Time = 10–20 min	Time = 20–50 min	Time = 55–60 min	
	Initial (time 0–10 min)	Ligand challenge at 10 min	Ligand washed out after 10 min incubation and measured cAMP 20–30 min post washing	cAMP following 500 nM α-MSH re-challenge at 55 min	
α-MSH, 200 nM	−	++++	−	++++	Full reversal of signal upon washing out
LY2112688, 100 nM	−	++++	−	++++	Full reversal of signal upon washing out
THIQ, 1 µM	−	++++	++	++++	Partial reversal upon washout
MT-II, 200 nM	−	++++	++++	++++	No reversal upon washout
RM-511, 100 nM	−	++++	++++	++++	No reversal upon washout

− represents a zero or basal level response, or response without any ligand exposure; ++ represents about half the maximal cAMP stimulation response; ++++ represents a 100% cAMP stimulation response.

**Table 4:** Data from Nickolls et al. [31] showing impaired internalization of MC4R by small molecule agonists as compared with the peptide agonists. The general structure of these small molecules is given directly below.

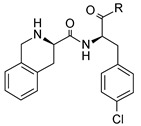					
**Ligand**	**R**	**MC4R Binding Ki (nM)**	**MC4R EC_50_ (nM)** ***(Intrinsic Activity)***		
			**cAMP Accumulation**	**Calcium Mobilization**	**Internalization**
α-MSH *	Acetyl-SYSMEHFRWGKPV-amide	50.1	21.4 *(100)*	129 *(100)*	28.8 *(100)*
β-MSH *	DEGPYRMEHFRWGSPPKD	18.6	11.2 *(83)*	174 *(94)*	43.7 *(118)*
γ-MSH *	YVMGHFRWDRFG	589	631 *(80)*	>1000 *(62)*	>1000 *(75)*
des-acetyl-α-MSH	SYSMEHFRWGKPV-amide	30.2	17.4 *(84)*	110 *(96)*	30.2 *(107)*
NDP-α-MSH	Acetyl-SYS(Nle)H(D-Phe)-RWGKPV-amide	3.98	1.38 *(112)*	120 *(80)*	7.24 *(89)*
THIQ	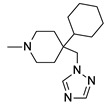	10.7	1.32 *(98)*	692 *(7)*	0.81 (29) **
NBI-55886	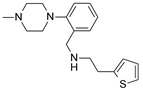	9.55	331 *(73)*	>1000 *(40)*	3.47 (16) **
NBI-56297	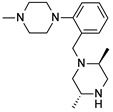	20	129 *(81)*	>1000 *(23)*	7.94 (−5) **
NBI-56453	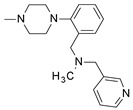	74.1	513 *(103)*	45.7 *(7)*	5.25 (27) **
NBI-58702	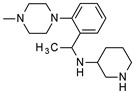	6.31	43 *(110)*	2.4 *(20)*	7.59 (38) **
NBI-58704	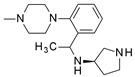	13.5	204 *(149)*	17.8 *(23)*	145 (34) **

* Endogenous melanocortin receptor-4 agonist. ** For all the small molecules, the intrinsic activity for internalization was significantly different and lower than that in the cAMP accumulation assay.

## References

[1] Marks D.L., Cone R.D. (2001). Central melanocortins and the regulation of weight during acute and chronic disease. Recent Prog. Horm. Res..

[2] Cone R.D. (1999). The central melanocortin system and its role in energy homeostasis. Ann. Endocrinol..

[3] Collet T.H., Dubern B., Mokrosinski J., Connors H., Keogh J.M., Mendes de Oliveira E., Henning E., Poitou-Bernert C., Oppert J.M., Tounian P. (2017). Evaluation of a melanocortin-4 receptor (MC4R) agonist (Setmelanotide) in MC4R deficiency. Mol. Metab..

[4] Clément K., Biebermann H., Farooqi I.S., Van der Ploeg L., Wolters B., Poitou C., Puder L., Fiedorek F., Gottesdiener K., Kleinau G. (2018). MC4R agonism promotes durable weight loss in patients with leptin receptor deficiency. Nat. Med..

[5] Balthasar N., Coppari R., McMinn J., Shun M., Liu S.M., Charlotte E., Lee C.E., Tang V., Kenny C.D., McGovern R.A. (2004). Leptin receptor signaling in POMC neurons is required for normal body weight homeostasis. Neuron.

[6] Lee Y.S. (2009). The Role of Leptin-Melanocortin System and Human Weight Regulation: Lessons from Experiments of Nature. Ann. Acad. Med. Singapore.

[7] Van der Klaauw A.A., Farooqi I.S. (2015). The hunger genes: Pathways to obesity. Cell.

[8] Van der Ploeg L.H.T., Martin W.J., Martin A.D., Nargund R.P., Austin C.P., Guan X.-M., Drisko J., Cashen D., Sebhat I., Patchett A.A. (2002). A role for the melanocortin 4 receptor in sexual function. Proc. Natl. Acad. Sci. USA.

[9] Martin W.J., MacIntyre D.E. (2004). Melanocortin receptors and erectile function. Eur. Urol..

[10] Kuo J.J., da Silva A.A., Tallam L.S., Hall J.E. (2006). Role of adrenergic activity in pressor responses to chronic melanocortin receptor activation. Hypertension.

[11] Greenfield J.R., Miller J.W., Keogh J.M., Henning E., Satterwhite J.H., Cameron G.S., Astruc B., Mayer J.P., Brage S., See T.C. (2009). Modulation of blood pressure by central melanocortinergic pathways. N. Engl. J. Med..

[12] Tao Y.-X. (2010). The melanocortin-4 receptor: Physiology, pharmacology, and pathophysiology. Endocr. Rev..

[13] MacNeil D.J., Howard A.D., Guan X.-M., Fong T.M., Nargund R.P., Bednarek M.A., Goulet M.T., Weinberg D.H., Strack A.M., Marsh D.J. (2002). The role of melanocortins in body weight regulation: Opportunities for the treatment of obesity. Eur. J. Pharmacol..

[14] Palucki B.L., Park M.K., Nargund R.P., Ye Z.X., Sebhat I.K., Pollard P.G., Kalyani R.N., Tang R., MacNeil T., Weinberg D.H. (2005). Discovery of (2S)-N-[(1R)-2-[4-cyclohexyl-4-[[(1,1-dimethylethyl)amino]carbonyl]-1-piperidinyl]-1-[(4-fluorophenyl)methyl]-2-oxoethyl]-4-methyl-2-piperazinecarboxamide (MB243), a potent and selective melanocortin subtype-4 receptor agonist. Bioorg. Med. Chem. Lett..

[15] Ujjainwalla F., Sebhat I.K. (2007). Small molecule ligands of the human melanocortin-4 receptor. Curr. Top. Med. Chem..

[16] Chen C., Jiang W., Tran J.A., Tucci F.C., Fleck B.A., Markison S., Wen J., Madan A., Hoare S.R., Foster A.C. (2008). Identification and characterization of pyrrolidine diastereoisomers as potent functional agonists and antagonists of the human melanocortin-4 receptor. Bioorg. Med. Chem. Lett..

[17] Krishna R., Gumbiner B., Stevens C., Musser B., Mallick M., Suryawanshi S., Maganti L., Zhu H., Han T.H., Scherer L. (2009). Potent and selective agonism of the melanocortin receptor 4 with MK-0493 does not induce weight loss in obese human subjects: Energy intake predicts lack of weight loss efficacy. Clin. Pharmacol. Ther..

[18] He S.W., Ye Z.X., Dobbelaar P.H., Sebhat I.K., Guo L.Q., Liu J., Jian T.Y., Lai Y.J., Franklin C.L., Bakshi R.K. (2010). Discovery of a spiroindane based compound as a potent, selective, orally bioavailable melanocortin subtype-4 receptor agonist. Bioorg. Med. Chem. Lett..

[19] Lansdell M.I., Hepworth D., Calabrese A., Brown A.D., Blagg J., Burring D.J., Wilson P., Fradet D., Brown T.B., Quinton F. (2010). Discovery of a selective small-molecule melanocortin-4 receptor agonist with efficacy in a pilot study of sexual dysfunction in humans. J. Med. Chem..

[20] Todorovic A., Haskell-Luevano C. (2005). A review of melanocortin receptor small molecule ligands. Peptides.

[21] Ericson M.D., Lensing C.J., Fleming K.A., Schlasner K.N., Doering S.R., Haskell-Luevano C. (2017). Bench-top to clinical therapies: A review of melanocortin ligands from 1954 to 2016. Biochim. Biophys. Acta Mol. Basis Dis..

[22] White W.B., Myers M.G., Jordan R., Lucas J. (2017). Usefulness of ambulatory blood pressure monitoring to assess the melanocortin receptor agonist bremelanotide. J. Hypertens..

[23] Clayton A.H., Althof S.E., Kingsberg S., DeRogatis L.R., Kroll R., Goldstein I., Kaminetsky J., Spana C., Lucas J., Jordan R. (2016). Bremelanotide for female sexual dysfunctions in premenopausal women: A randomized, placebo-controlled dose-finding trial. Womens Health (Lond).

[24] Kievit P., Halem H., Marks D.L., Dong J.Z., Glavas M.M., Sinnayah P., Pranger L., Cowley M.A., Grove K.L., Culler M.D. (2013). Chronic treatment with a melanocortin-4 receptor agonist causes weight loss, reduces insulin resistance, and improves cardiovascular function in diet-induced obese rhesus macaques. Diabetes.

[25] Van der Ploeg L.H.T. (2012). Rhythm Pharmaceuticals, Boston, MA, USA.

[26] Yang L.K., Tao Y.X. (2017). Biased signaling at neural melanocortin receptors in regulation of energy homeostasis. Biochim. Biophys. Acta Mol. Basis Dis..

[27] Büch T.R., Heling D., Damm E., Gudermann T., Breit A. (2009). Pertussis toxin-sensitive signaling of melanocortin-4 receptors in hypothalamic GT1-7 cells defines agouti-related protein as a biased agonist. J. Biol. Chem..

[28] Kumar K.G., Sutton G.M., Dong J.Z., Roubert P., Plas P., Halem H.A., Culler M.D., Yang H., Dixit V.D., Butler A.A. (2009). Analysis of the therapeutic functions of novel melanocortin receptor agonists in MC3R- and MC4R-deficient C57BL/6J mice. Peptides.

[29] Shinyama H., Masuzaki H., Fang H., Flier J.S. (2003). Regulation of melanocortin-4 receptor signaling: Agonist-mediated desensitization and internalization. Endocrinology.

[30] Mayer J.P., Hsiung H.M., Flora D.B., Edwards P., Smith D.P., Zhang X.Y., Gadski R.A., Heiman M.L., Hertel J.L., Emmerson P.J. (2005). Discovery of a beta-MSH-derived MC-4R selective agonist. J. Med. Chem..

[31] Nickolls S.A., Fleck B., Hoare S.R., Maki R.A. (2005). Functional selectivity of melanocortin 4 receptor peptide and nonpeptide agonists: Evidence for ligand-specific conformational states. J. Pharmacol. Exp. Ther..

[32] Haskell-Luevano C., Monck E.K. (2001). Agouti-related protein functions as an inverse agonist at a constitutively active brain melanocortin-4 receptor. Regul. Pept..

[33] Nijenhuis W.A., Oosterom J., Adan R.A. (2001). AgRP(83-132) acts as an inverse agonist on the human melanocortin-4 receptor. Mol. Endocrinol..

[34] Molden B.M., Cooney K.A., Wes T.K., Van Der Ploeg L.H., Baldini G. (2015). Temporal cAMP Signaling Selectivity by Natural and Synthetic MC4R Agonists. Mol. Endocrinol..

[35] Granell S., Molden B.M., Baldini G. (2013). Exposure of MC4R to agonist in the endoplasmic reticulum stabilizes an active conformation of the receptor that does not desensitize. Proc. Natl. Acad. Sci. USA.

[36] Huang H., Wang W., Tao Y.X. (2017). Pharmacological chaperones for the misfolded melanocortin-4 receptor associated with human obesity. Biochim. Biophys. Acta Mol. Basis Dis..

[37] Gao Z., Lei D., Welch J., Le K., Lin J., Feng S., Duhl D. (2003). Agonist-dependent internalization of the human melanocortin-4 receptors in human embryonic kidney 293 cells. J. Pharm. Exp. Ther..

[38] Cai M., Varga E.V., Stankova M., Mayorov A., Perry J.W., Yamamura H.I., Trivedi D., Hruby V.J. (2006). Cell signaling and trafficking of human melanocortin receptors in real time using two-photon fluorescence and confocal laser microscopy: Differentiation of agonists and antagonists. Chem. Biol. Drug Des..

[39] Li Y.Q., Shrestha Y., Pandey M., Chen M., Kablan A., Gavrilova O., Offermanns S., Weinstein L.S. (2016). G(q/11)α and G(s)α mediate distinct physiological responses to central melanocortins. J. Clin. Investig..

[40] Ghamari-Langroudi M., Digby G.J., Sebag J.A., Millhauser G.L., Palomino R., Matthews R., Gillyard T., Panaro B.L., Tough I.R., Cox H.M., Denton J.S. (2015). G-protein-independent coupling of MC4R to Kir7.1 in hypothalamic neurons. Nature.

[41] Vongs A., Lynn N.M., Rosenblum C.I. (2004). Activation of MAP kinase by MC4-R through PI3 kinase. Regul. Pept..

[42] Kuo J.J., da Silva A.A., Hall J.E. (2003). Hypothalamic melanocortin receptors and chronic regulation of arterial pressure and renal function. Hypertension.

[43] Ni X.-P., Butler A.A., Cone R.D., Humphreys M.H. (2006). Central receptors mediating the cardiovascular actions of melanocyte stimulating hormones. J. Hypertension.

[44] Humphreys M.H., Ni X.-P., Pearce D. (2011). Cardiovascular effects of melanocortins. Eur. J. Pharmacol..

[45] Hill C., Dunbar J.C. (2002). The effects of acute and chronic alpha melanocyte stimulating hormone (alphaMSH) on cardiovascular dynamics in conscious rats. Peptides.

[46] Matsumura K., Tsuchihashi T., Abe I., Iida M. (2002). Central alpha-melanocyte-stimulating hormone acts at melanocortin-4 receptor to activate sympathetic nervous system in conscious rabbits. Brain Res..

[47] Nordheim U., Nicholson J.R., Dokladny K., Dunant P., Hofbauer K.G. (2006). Cardiovascular responses to melanocortin 4-receptor stimulation in conscious unrestrained normotensive rats. Peptides.

[48] Trivedi P., Jiang M., Tamvakopoulos C.C., Shen X., Yu H., Mock S., Fenyk-Melody J., Van der Ploeg L.H.T., Guan X.M. (2003). Exploring the site of anorectic action of peripherally administered synthetic melanocortin peptide MT-II in rats. Brain Res..

[49] Lotta L.A., Mokrosin’ski J., de Oliveira E.M., Li C., Sharp S.J., Luan J., Brouwers B., Ayinampudi V., Bowker N., Kerrison N. (2019). Human Gain-of-Function *MC4R* Variants. Cell.

